# Onchocerciasis: the beginning of the end?

**Published:** 2019-02-10

**Authors:** Adrian Hopkins

**Affiliations:** 1 Consultant. Former Director of the Mectizan Donation Programme. Gravesend, UK.

**With a concerted effort, onchocerciasis could be the first blinding disease to be eradicated.**

Onchocerciasis causes skin and eye disease, visual impairment and neurological problems. It is mostly found in Africa, but also in Latin America and Yemen. The common name, ‘river blindness,’ gives a good indication where the disease can be found: the vector of the parasite, a small black fly of the *Simulum* species, breeds in rivers where there is turbulence in the water, such as rapids, or where the flow is disturbed by overhanging vegetation.

The medicine first used to treat onchocerciasis was diethylcarbamazine. Unfortunately, it was associated with severe inflammation and deterioration of vision and so was eventually contraindicated.

In the 1970s, efforts were undertaken to prevent onchocerciasis by controlling the black fly that spread the disease. This was done by spraying larvicide over black fly breeding sites, which killed the larvae before they could develop into adult flies. This proved to be successful at stopping transmission and became the basis of the Onchocerciasis Control Programme (OCP) in 11 countries in West Africa.

A paradigm shift in the management of onchocerciasis occurred when MSD (known as Merck & Co Inc. in the USA and Canada) announced that they would donate a new drug, Mectizan (ivermectin MSD), that was safe to use and had been shown to be effective.

In October 1987, MSD agreed to provide the drug free of charge to as many people as needed, for as long as it was needed. This led to the expansion of control efforts to other countries in Africa, Latin America and Yemen.

At the end of 1995, the African Programme for Onchocerciasis Control (APOC) was created. New mapping procedures were developed and mass drug administration (MDA) with ivermectin was carried out in heavily infected areas. A novel approach to distribution, known as community-directed treatment with ivermectin (CDTI), put communities in charge of deciding how and when distribution would take place. APOC was very successful as a control programme and was even expanded into countries that were experiencing conflict.

A further paradigm shift occurred when research showed that ivermectin alone could interrupt the transmission of infection in Africa. This had already been demonstrated in Latin America, where the disease occurs in small, isolated areas, or foci. Interruption of transmission is very important if onchocerciasis is to be eliminated rather than controlled as a public health problem.

## What do we need to do to eliminate onchocerciasis globally?

Countries are being urged to do the following:

- Set up national committees as part of their neglected tropical disease (NTD) programmes to review each focus of infection in detail and decide on the best strategy for individual foci.
- Expand mapping to include all potential areas of disease, not just heavily infected areas, to decide where treatment is needed. This will be done using a new mapping technique called onchocerciasis elimination mapping (OEM).
- Build laboratory capacity and implement monitoring and evaluation through epidemiological and entomological surveys.
- Continue with advocacy as the disease gradually disappears and is no longer a major issue in endemic countries.

Alternative, or additional, strategies must be explored:

- There is a need to evaluate the best use of ivermectin, e.g., conducting MDA twice (or even more times) per year.
- New drugs, especially those that can kill the adult parasite worm, would greatly speed up elimination
- Vector control using insecticides or, more. appropriately, removing vegetation from river banks, can increase the impact of MDA.

Many countries in Africa may be able to stop treatment by 2020. Can treatment be stopped everywhere by 2025? With concerted efforts, the answer could be yes; however, if the effort is not made, recurrence of the disease becomes a very real possibility.

**Figure:**
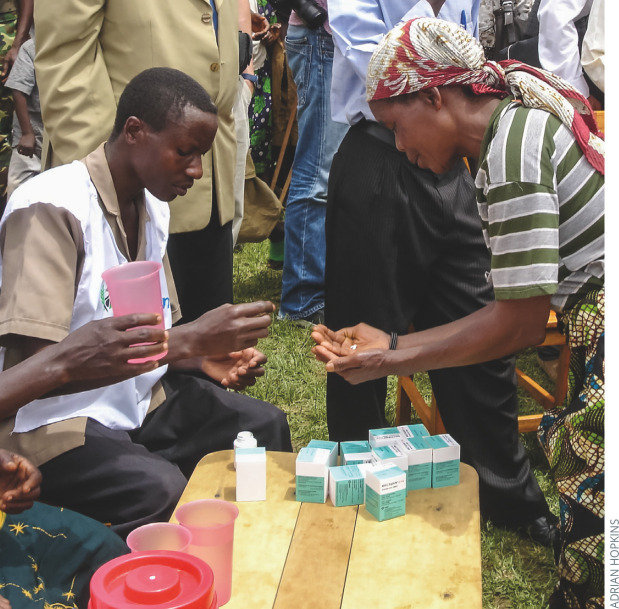
Community distribution of ivermectin (Mectizan^®^). **BURUNDI**

## Useful websites

1. For technical guidance Onchocerciasis: Guidelines for stopping mass drug administration and verifying elimination of human onchocerciasis **http://apps.who.int/iris/bitstream/handle/10665/204180/9789241510011_eng.pdf**
2. For advocacy River Blindness: The beginning of the end **https://mectizan.org/wp-content/uploads/2018/11/Onchocerciasis-Advocacy-Document.pdf**
3. For details on Mectizan: **https://mectizan.org**
